# Flexible Temperature Sensor Integrated with Soft Pneumatic Microactuators for Functional Microfingers

**DOI:** 10.1038/s41598-019-52022-x

**Published:** 2019-10-30

**Authors:** Satoshi Konishi, Akiya Hirata

**Affiliations:** 1 Department of Mechanical Engineering, College of Science and Engineering, Ritsumeikan University, Kusatsu, 525-8577 Japan; 2 Graduate Course of Science and Engineering, Ritsumeikan University, Kusatsu, 525-8577 Japan; 3 Ritsumeikan Global Innovation Research Organization, Ritsumeikan University, Kusatsu, 525-8577 Japan

**Keywords:** Electrical and electronic engineering, Mechanical engineering

## Abstract

The integration of a flexible temperature sensor with a soft microactuator (a pneumatic balloon actuator) for a functional microfinger is presented herein. A sensor integrated with a microactuator can actively approach a target for contact detection when a distance exists from the target or when the target moves. This paper presents a microfinger with temperature sensing functionality. Moreover, thermocouples, which detect temperature based on the Seebeck effect, are designed for use as flexible temperature sensors. Thermocouples are formed by a pair of dissimilar metals or alloys, such as copper and constantan. Thin-film metals or alloys are patterned and integrated in the microfinger. Two typical thermocouples (K-type and T-type) are designed in this study. A 2.0 mm × 2.0 mm sensing area is designed on the microfinger (3.0 mm × 12 mm × 400 μm). Characterization indicates that the output voltage of the sensor is proportional to temperature, as designed. It is important to guarantee the performance of the sensor against actuation effects. Therefore, in addition to the fundamental characterization of the temperature sensors, the effect of bending deformation on the characteristics of the temperature sensors is examined with a repeated bending test consisting of 1000 cycles.

## Introduction

This paper describes the integration of a sensor into a functional soft microfinger for a microhand robot operating in a small space. In this study, a soft microfinger was designed for a microhand robot of a teleoperated haptic robot system, which works in combination with an operator-controlled master device. The slave microhand is teleoperated in accordance with the motion of an operator through the master device. In the teleoperated haptic robot system, sensors integrated into the microfingers are expected to detect tactile senses. Then, the detected information is transmitted to the master device for sense presentation.

Various teleoperated manipulators have been developed in robotics for operation in inaccessible environments^1^. Minimally invasive medicine requires small and safe manipulators to improve operations in limited spaces, such as an abdominal cavity^2^. In biotechnology, especially in cellular engineering for regenerative medicine, it is important to handle fragile cells or tissues without introducing damage^3,4^. In one study, a manipulator for cellular aggregates^3^ was made of a soft material and was driven by pneumatic pressure. Biomedical applications require small, soft, and safe manipulators. MEMS or micromachine technology have enabled the fabrication of small devices; therefore, various soft polymer-based microactuators have been developed due to advances in such technology^5–8^. For instance, dielectric elastomer, ferroelectric polymers, and ionic polymer-metal composites have been discussed for artificial muscle applications. Poly(styrene-alt-maleimide)-incorporated poly(vinylidene fluoride), an electro-active polymer, has also been proposed for use in artificial muscles^6^. In contrast to many applications that use electrical energy, biomedical applications often use pneumatic force for actuation. For instance, balloon catheters are driven by pneumatic pressure. In addition, elastic inflatable actuators have been developed in various ways^9,10^. Soft robotics is one of the most attractive applications of elastic inflatable actuators. In the last ten years, numerous soft robotics have been reported. For example, various designs of soft robots actuated by pneumatic inflation have been reported^11,12^. In addition to soft materials for structures, pneumatic actuation can improve the compliance of microactuators.

One of the authors of this study have been continuously developing pneumatic balloon actuators (PBAs) since 1999^13^. PBAs can be categorized as inflatable microactuators. Bending-type PBAs contain a cavity between two bonded films (balloon and base films) that exhibit different mechanical properties. The typical PBA design uses polydimethylsiloxane (PDMS) films of different thicknesses and/or stiffnesses^14^. Thin and elastic PDMS films deform more than thick and stiff PDMS films; therefore, the PBA structure preferentially bends toward the thick and stiff film side. The main design parameters of the PBA are thickness and stiffness of films. Moreover, the lengths of edges of the balloon in the rectangular shape are important. The principle of the bending motion is explained by the pressure-dependent stiffness change in the balloon film. The thin and elastic balloon is first inflated, and its stiffness increases. In general, the stress-strain curve of rubbers shows a steep rise after a gentle increase. The steep rise of the stress-strain curve is regarded as the stiffness change. As a result, the balloon film becomes stiffer than the base film. The PBA bends toward the thick and stiff film side due to its pulling force. The soft microfinger in this study is made of a polymer and is driven by PBAs as artificial muscle. The typical dimensions of previously reported microfingers are 1.0 mm × 7.0 mm × 100 μm, and the plane shape of each PBA is a rectangle with dimensions of 300 μm × 1.5 mm. The reported microfinger is designed for manipulation of fish roe (1.0 mm in diameter). The rectangular shape of the PBAs preferentially generates anisotropic deformation along the longitudinal axis. The deformation of the inflated balloon is examined based on plate structure theory^15^. The deformation of a plate fixed at all edges depends on the aspect ratio of the lengths of the shorter and longer edges. The deformation of the plate increases with the aspect ratio and saturates when the aspect ratio reaches 2.0. The bending moment at the edge also similarly depends on the aspect ratio. The bending-type PBAs are arranged according to the intended degrees of freedom of the microfinger. Herein, two PBAs in series are designed as knuckle joints to provide the intended degrees of freedom of the microfinger considering the aspect ratio of the balloon. Specifically, a rectangular shape for which the longer edge is between five and six times longer than the shorter edge is designed for the PBAs in this study. Sequentially, sensors were designed for the PBA of the microfingers. Microfinger motion detection is necessary for motion control. A fluid-resistive strain sensor with a microchannel was integrated with a PBA via batch fabrication to detect the bending motion of a microfinger. Both PBAs and fluid-resistive strain sensors can be formed by spaces such as cavities and channels between two PDMS films. Fluid resistance changes depend on the deformation of the microchannel, and bending motion can be detected by the changes in fluid resistance. Furthermore, the authors utilized liquid metal in the strain sensor for microfinger motion detection. A recent study reported a strain sensor containing liquid metals, including Galinstan^16^, i.e. instead of a fluid. Galinstan was injected and filled in the microchannel of a fluid-resistive sensor. The electrical resistance of the liquid metal in the microchannel changes due to the deformation of the microfinger. By replacing a fluid with a liquid metal in the microchannel, the electrical signals can be addressed directly. The function of tactile sensing using this type of strain sensor has also been studied by analyzing the influence of the force from a contacted object on the bending motion of a microfinger. Tactile sensing information is available for tactile sense presentation through the combination of human interface devices.

Subsequently, this paper focuses on a temperature sensor integration to a microfinger as a part of the whole research aiming at the construction of the teleoperated haptic robot system because temperature sensing is one of major sensory functions of human fingers. Sensors integrated into microfingers can provide tactile sense detection for functional microfingers. Moreover, microfinger actuation enables active sensing of targets. Active sensing is effective when a distance exists between the sensor and the target or when the target moves. Our previous report evaluated the influence of the gap distance between a sensor and a target in temperature sensing^17^. Thermistors and thermocouples are very popular devices for temperature detection^18–22^. Thermistors detect temperature through temperature-dependent changes in electrical resistance. In contrast, thermocouples detect temperature through the Seebeck effect. When two different conductive materials are connected to each other and a temperature gradient exists across the connected structure, the Seebeck effect generates a potential difference between both ends of the two materials. This potential difference is generated due to the diffusion of charge carriers along a temperature gradient when one side of the connected structure is heated or cooled. As a first trial, we reported a discrete type of integration of commercialized thermistors on the tip of a microfinger^17^. In the first trial, a thermistor was embedded at the tip of a microfinger and was interconnected with thin-film electrode wires of titanium and gold. This study aims to develop functional microfingers by utilizing the technology of micro electromechanical systems (MEMS) in the form of microsensors and microactuators. It is important to improve manufacturing efficiency by minimizing assembly steps. In the MEMS field, platinum resistance temperature sensors have been reported as having high sensitivity^23–25^. Thermistors using platinum, which is corrosion resistant, are highly superior to other thermistors from the viewpoint of oxidation resistance; however, platinum-based thermistors are expensive. Moreover, to guarantee safety, the microfingers are not reused after being implemented in biomedical applications. This study focuses on the integration of a thin-film thermocouple into the microfinger with minimum assembly. The selected thermocouple has good responsiveness and an inexpensive production cost. Moreover, the selected thermocouple can be fabricated with minimum assembly by the simple combination of thin-film metals or alloys.

Deformation owing to the bending actuation of a microfinger causes strain in the structure of the microfinger. The mechanical characteristics, especially the Young’s modulus of PDMS, are more important than those of other conventional materials used for sensors and actuators^26^. Strain analyses of metal films on PDMS have shown that research has overcome the problems related to differences in the Young’s modulus between metals and PDMS^27^. PBAs use inflating balloons, which generate internal stresses in addition to the stresses by bending motions. The effect of internal stresses on the performance of strain sensors has been previously investigated and reported^28^. The strain from actuation affects not only the performance of the strain sensor integrated into the microfinger but also the characteristics of the peripheral interconnection wires. Electrical resistance changes depending on the strain. It is important for thermocouples composed of thin-film metals or alloys to perform reliability against such actuation effects. We performed a repeated bending test to estimate the characteristics of thermocouples using thin-film metals or alloys integrated in a deformable polymer. The actuation dependent behavior of these sensors will be reported and investigated herein. Furthermore, many studies on stretchable electronics have been reported for the application of flexible devices, such as wearable devices^29–37^. It becomes increasingly important to focus on the flexibility or softness for wearable or portable products, in terms of high human affinity. Wavy and stretchable Si electronics on elastomeric substrates have been reported for stretchable devices^29,34^. Polymer-based organic photonic devices have been demonstrated by combining highly flexible conducting polymers, semiconducting polymers and thin metal layers^28,30^. These smart designs would be effective in improving the robustness of sensors integrated into microfingers in the future, regardless of the sufficient reliability demonstrated by the current device in our application.

## Results

### Fabrication results of integrating the thermocouples into soft microfingers

**Figure 1:**
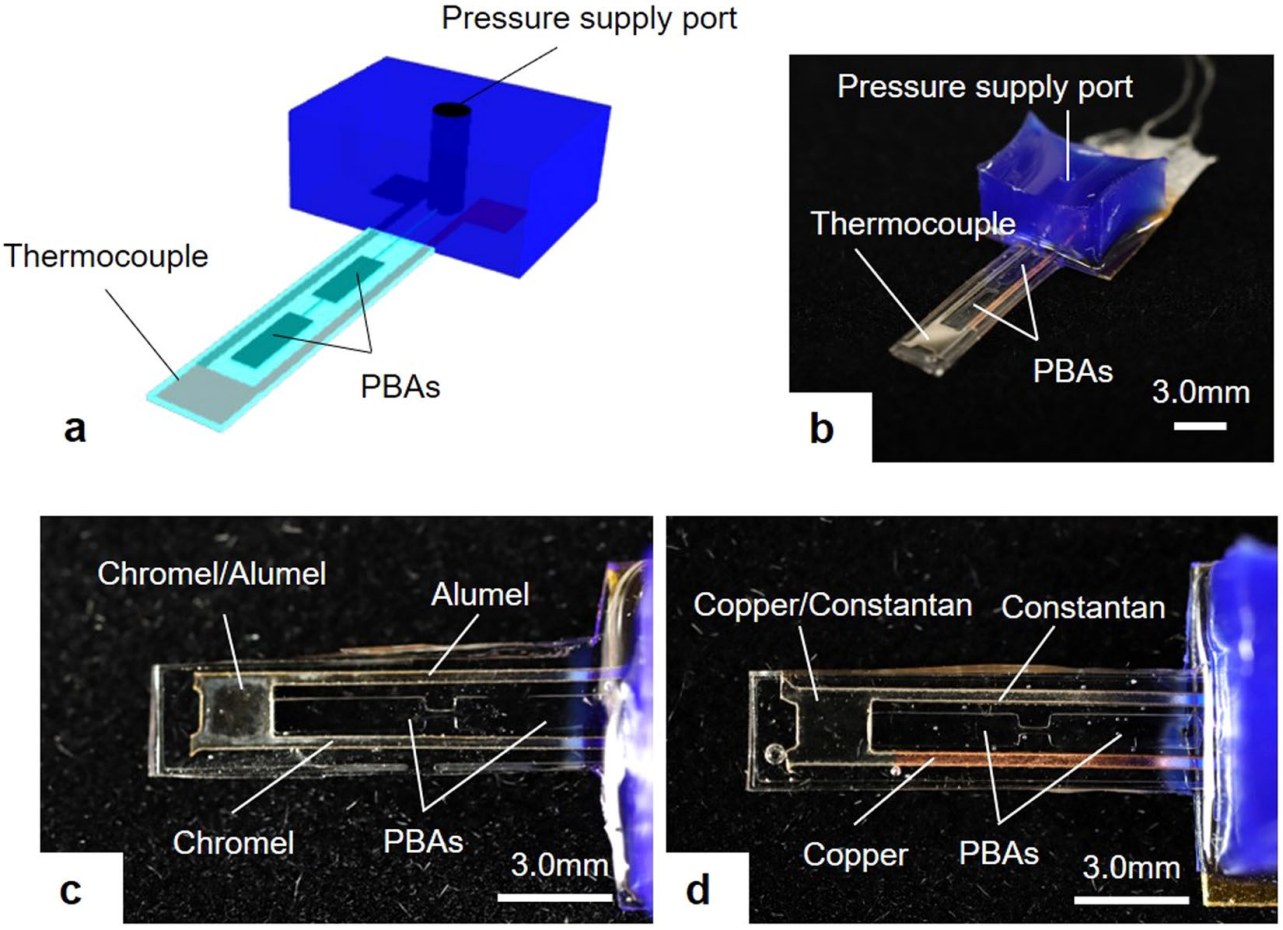
Fabrication results of thermocouple integrated into the soft microfinger. (**a**) Schametic drawings of the thermocouple. (**b**) A photograph of fabricated thermocouple into the soft microfinger. Two PBAs are arranged in series along the center of the microfinger, and the sensor was integrated at the tip of the microfinger. Each balloon was 800 µm × 4.0 mm. The temperature sensing part was 2.0 mm × 2.0 mm. **(c)** A magnified top view of the device integrated with K-type thermocouple. The chromel and alumel for the thermocouple were formed on a polyimide film. The interconnection wires of width 400 μm were formed from the sensing part to the root of the microfinger. **(d)** T-type thermocouple consisted of copper and constantan. Dimensions were same as the K-type sensor.

Figure 1 shows the fabrication results of a thermocouple integrated into the soft microfinger. Schematic drawings are shown in Fig. 1a. A photograph of the fabrication results is shown in Fig. 1b. A flexible temperature sensor was designed to be integrated into a soft microfinger that was fabricated using PDMS and driven by a PBA. Previously, we reported the assembly of a commercial thermistor as a first preliminary trial. In the previous work, the thermistor (500 μm × 1000 μm × 150 μm, 10KC05-1005LD, SEMITEC Co.) was embedded at the tip of a microfinger and interconnected with thin-film titanium and gold electrode wires (300 nm thick) on the microfinger. In contrast to the previous report, which used an assembled commercial thermistor, this study designs and integrates a thin-film thermocouple into a microfinger. Two PBAs (800 μm × 4.0 mm) are connected in series for the microfinger (3.0 mm × 12 mm × 400 μm) in this study.

A thermocouple is composed of a junction of two dissimilar metals or alloys. The dissimilar films are patterned and overlapped at the junction. A thermocouple generates a voltage proportional to the difference in temperature between the measurement junction and the reference junction based on the Seebeck effect. The voltage at the measurement junction depends on the temperature when the reference temperature is fixed. A K-type thermocouple was designed first, as this type of thermocouple is the most popular. A T-type thermocouple was also designed, as this type of thermocouple is the most suitable sensor for the soft microfinger made of PDMS. The K-type sensor consists of chromel and alumel, whereas the T-type thermocouple employs copper and constantan. Figure 1c,d show magnified top views of the K-type and T-type sensors, respectively. Electrode wires of two metals or alloys were overlapped and connected at the measurement junction on the microfinger. The dimensions were the same for both types of sensors. The junction part of the two metals or alloys was fabricated at the tip of the microfinger. The PBAs were arranged along the center of the microfinger, and the sensor was integrated at the tip of the microfinger. The two different materials for the thermocouple were deposited on a patterned polyimide structure. The dimensions of the temperature-sensing part, where the thin-film metals or alloys were overlapped, were 2.0 mm × 2.0 mm. The interconnection wires with a width of 400 μm were formed from the sensing part to the root of the microfinger. The dimensions of temperature sensors were decided in consideration of the electrical resistance of the thermocouple and the whole structure of the microfinger. A similar approach was used to integrate both K-type and T-type sensors into the microfinger structure. Moreover, the performance of K-type and T-type sensors (i.e., the candidates in this study) will be compared.

### Characterization of thermocouples

**Figure 2:**
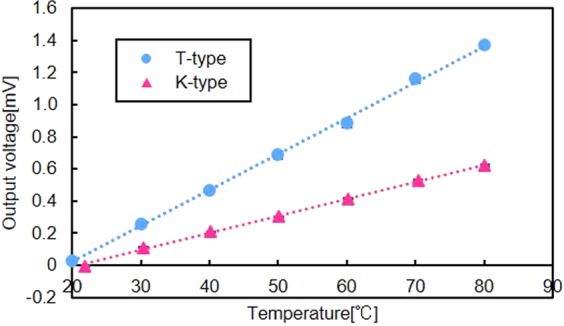
Characterization of the fabricated thermocouples. Characterization of the output voltage from the thermocouples with respect to the temperature for K-type and T-type sensors. The standard error is used in Fig. 2. The output voltage changed proportionally to the temperature at the junction. The output voltage increased linearly with respect to the temperature. The thermoelectromotive force of K-type sensor was approximately 10.6 μV/°C. The thermoelectromotive force of the fabricated T-type sensor was estimated as 22.3 μV/°C.

The fabricated K-type and T-type thermocouples were characterized, and the results are shown in Fig. 2. Figure 2 shows the output voltage from the thermocouples with respect to temperature. The standard error is used in Fig. 2. The output voltage changed proportionally to the temperature at the junction of the two materials. In the experiment, the temperature changed from 20 °C to 80 °C, and the reference temperature was set to 20 °C. The output voltage increased linearly as the temperature increased, and. The thermoelectromotive force of the fabricated K-type sensor was estimated as 10.6 μV/°C, where the thermoelectromotive force of the T-type sensor was approximately 22.3 μV/°C.

### Bending motion of the soft microfinger and its effect on the sensor characteristics

**Figure 3:**
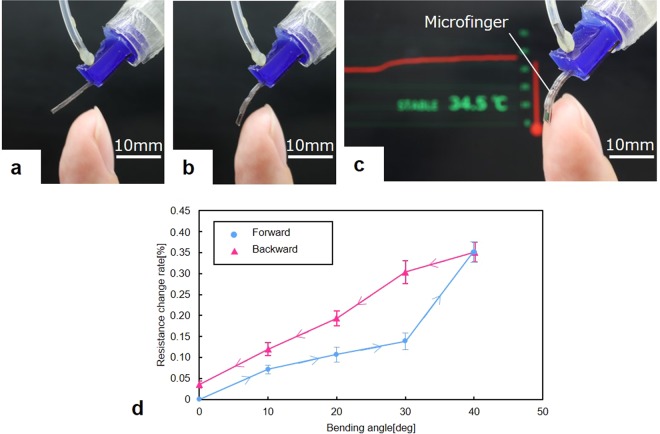
Active sensing by bending microfinger and the electrical resistance change rate of thermocouple depending on the bending angle of microfinger. (**a**–**c**) The soft microfinger integrated with thermocouple was bent by the microfinger. The microfinger approached and touched a human finger. The detected temperature of the human finger is displayed in the monitor. (**d**) The effect of the bending motion on the characteristics of the thermocouple was evaluated at room temperature. The standard error is used in (**d**). The resistance change rate after 50 successive bending motions was evaluated. The electrical resistance increased by approximately 0.35% of the initial value when the bending angle was 40°. The resistance change rate according to the bending angle showed hysteresis characteristics.

The soft microfinger integrated with the thermocouple was bent by the microfinger, as shown in Fig. 3a–c. Furthermore, a 70 kPa pressure was applied to the PBA, which bent the PBA by 40° and generated a 0.65 mN force. The microfinger with the T-type thermocouple approached and touched a human finger. The detected temperature of the human finger is displayed in the monitor in Fig. 3c. The temperature of the human fingertip was confirmed by a commercial sensor at the same time. The detected signal is considerably affected by the gap distance between a sensor and a target in temperature sensing according to our previous report^17^. It was reported that contact sensing was important for a reliable measurement. The active sensing by the microfinger enables contact sensing. The influence of the PDMS structure between a sensor and a target was also evaluated in the report. No substantial influence was observed in this evaluation because of the thermal conduction characteristics of PDMS, which exhibited higher thermal conductivity than general organic rubbers. A sensor moved by a PBA can actively approach a target for contact detection when a distance exists from the target or when the target moves. This method is effective for active sensing against pulsating targets such as a living body. Furthermore, the electrical resistance of thermocouple including the interconnection wires was evaluated in accordance with the bending angle of the microfinger at room temperature (Fig. 3d). Figure 3d shows the resistance change rate after 50 successive bending motions. The standard error is used in Fig. 3d. The cross-sectional dimensions of the interconnection wire were 400 µm × 0.15 µm, and the length of the interconnection wire was approximately 20 mm. The electrical resistance increased when the microfinger was bent through pressurization. As a result, the electrical resistance increased by approximately 0.35% of the initial value, when the microfinger was bent by 40° at the tip. The resistance change rate according to the bending angle showed hysteresis characteristics. The resistance did not return to the initial value but slightly increased when the microfinger returned to the initial position.

**Figure 4:**
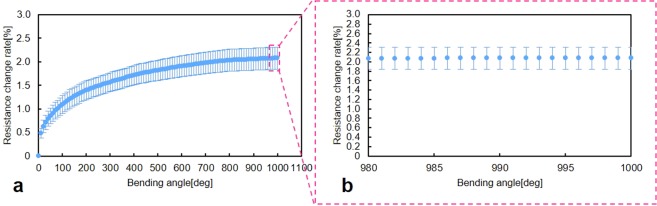
The repeated bending test. The electrical properties of the metals or alloys on a deformable PDMS structure was estimated through the repeated bending test at room temperature. The T-type thermocouple was evaluated when the microfinger was repeatedly bent by 40°. The standard error is used in Fig. 4. (**a**) The change rate of electrical resistance of the sensor according to the number of bending motions up to 1000 bending cycles. (**b**) The characteristics of the sensor at a high number of bending cycles are magnified.

A repeated bending test was executed to examine the electrical properties of the metals or alloys on a deformable PDMS structure at room temperature (Fig. 4). The microfinger was bent by 40°. The T-type (i.e., copper and constantan) thermocouple was evaluated through a repeated bending test. Figure 4a shows the change rate of electrical resistance of the sensor according to the number of bending motions up to 1000 bending cycles. The characteristics of the sensor at a high number of bending cycles are magnified in Fig. 4b. The standard error is used in Fig. 4.

## Discussion

The soft microfinger was designed as a disposable device for safety considerations in biomedical applications. Consequently, a thin-film thermocouple was chosen for this study as the temperature sensor integrated into the microfinger. The thermocouple exhibits the advantages of good responsiveness and an inexpensive production cost. The K-type and T-type thermocouples were successfully integrated into the microfinger (Fig. 1). Other types of thermocouples using precious metals, such as platinum and rhodium, are expensive. The K-type sensor consists of chromel and alumel, whereas the T-type thermocouple employs copper and constantan. The measurement ranges of the K-type and -T thermocouples are −200 to 1000 °C and −200 to 300 °C, respectively. The K-type thermocouple is the most popular thermocouple and exhibits good linearity. In terms of the thermoelectromotive force, T-type sensor is higher than that of K-type due to the physical property. The T-type thermocouple has good uniformity and shows high precision in rather low temperature range (−200 to 300 °C). PDMS, which is the primary structural material for the microfinger, has an upper operational temperature limit of at least 200 °C. It has been reported that the substantial thermal degradation of PDMS occurs at approximately 400 °C^38^. The required temperature range was assumed to be less than 50 °C for most biomedical applications. Both sensors exhibited good linearity in this range and could operate in the temperature range of the microfinger in biomedical applications. The thermoelectromotive force of the integrated T-type sensor was 22.3 μV/°C, whereas that of the K-type sensor was 10.6 μV/°C. The T-type sensor showed higher thermoelectromotive force as designed. This study could demonstrate the integration of both K-type and T-type thin-film thermocouples into soft microfingers for active temperature sensing with minimum assembly.

The mechanical properties of each material are important for designing soft sensors and actuators. The Young’s modulus of copper, constantan, chromel and alumel are between 100 and 200 GPa. All materials for the thermocouple in this study exhibit similar Young’s moduli, whereas the Young’s moduli of polyimide and  PDMS are reported to be around 5 GPa and  less than 1 MPa, respectively. PDMS is much more flexible and deformable than the metals and alloys used for thermocouples. It is important that metals or alloys can operate reliably in the deforming PDMS structure of a bending PBA. Wavy and stretchable Si electronics on elastomeric substrates^29,34^ and polymer-based organic photonic devices^28,30^ have been studied for stretchable electronics. Moreover, liquid metals have been studied as attractive materials for soft electronic conductors in this field^16,28,36,37^. These smart designs might be effective for stress relaxation techniques; however, additional processes are required to implement such designs. The requirements for stretchable structures are more challenging than those for the simple bending structure of the microfinger in this study.

As shown in Fig. 3d, the electrical resistance of the thermocouple increased and did not return to the initial value when the microfinger was bent by 40°. The resistance change rate according to the bending angle exhibited hysteresis characteristics. We prioritized the investigation of this phenomenon before considering potential problems. The electrical properties of the metals or alloys for the T-type thermocouple in the deformable PDMS structure were estimated through a repeated bending test at room temperature (Fig. 4). The resistance change rate in Fig. 4a was calculated using the resistance value after each bending motion. Figure 3d shows the transient behavior of the resistance of the repeated fiftieth times of bending motion. The electrical resistance increased according to the number of bending cycles and tended to converge to a certain value, as shown in Fig. 4a. The change rate of electrical resistance tended to converge to less than 3.0%. The overall change rate does not considerably affect most of our applications, whereas further microscopic investigation provides another interesting piece of information. We discovered that the current simple thermocouple design can be used in deformable PDMS structures for microfingers by understanding the characteristics of the change rate of electrical resistance based on the actuation.

## Methods

### Fabrication process of integrating the thermocouple into microfingers

**Figure 5:**
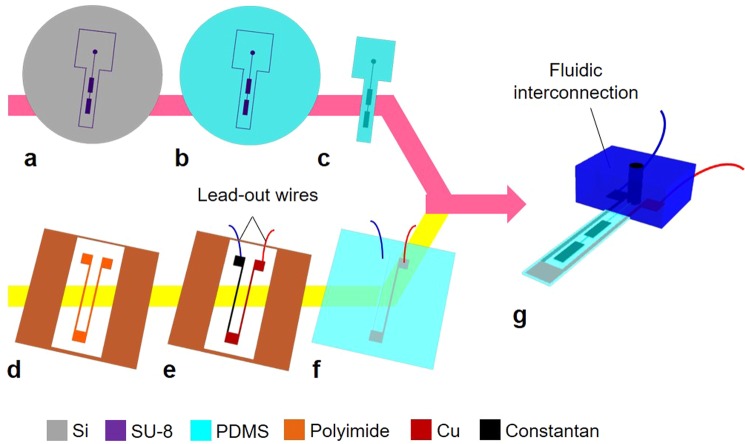
Fabrication process. The thermocouple was integrated into the microfinger as follows. (**a**–**c**) 70-µm-thick PDMS film with a cavity for PBA was fabricated by molding PDMS using patterns of negative photoresist SU-8 on a substrate. **(d)** the 50-µm-thick polyimide film was cut into the shape of a sensor and interconnection wires by CO_2_ laser. **(e)** The thermocouple structure was formed by the deposition of metals or alloys on the patterned polyimide film. **(f)** The thermocouple structure was placed on 100-µm-thick PDMS film and then coated by PDMS layer (200-µm-thick). **(g)** Finally, the PDMS film with the balloon cavity was released from the substrate and bonded onto the thermocouple structure. The fluidic interconnection was also implemented.

The thermocouple was integrated into the microfinger as shown in Fig. 5. 70-µm-thick PDMS (Silpot 184, Dow Corning Inc.) film with a cavity for PBA was fabricated by molding PDMS using patterns of negative photoresist SU-8 (MicroChem Co.) on a substrate (Fig. 5a–c). The prepolymer of PDMS and curing agent were mixed with 8:1. In parallel, the 50-µm-thick polyimide film was cut into the shape of a sensor and interconnection wires by CO_2_ laser (Fig. 5d). The thermocouple structure was formed by the deposition of metals or alloys on the patterned polyimide film (Fig. 5e). In the case of T-type thermocouple, the copper and constantan thin-films were sequentially deposited by evaporation over the polyimide structure. A lift-off process using a polyimide stencil mask was used to pattern thin-films of copper and constantan. Lead-out wires were implemented in advance to the following PDMS coating (Fig. 5e). The thermocouple structure was placed on 100-µm-thick PDMS film (12: 1 in mixture ratio of the prepolymer and curing agent) and then coated by 200-µm-thick PDMS layer (12: 1 in mixture ratio) (Fig. 5f). Finally, the PDMS film with the balloon cavity was released from the substrate and bonded onto the thermocouple structure using vacuum ultraviolet light. In addition, the fluidic interconnection was implemented to complete the functional microfinger (Fig. 5g).

### Experimental setup

**Figure 6:**
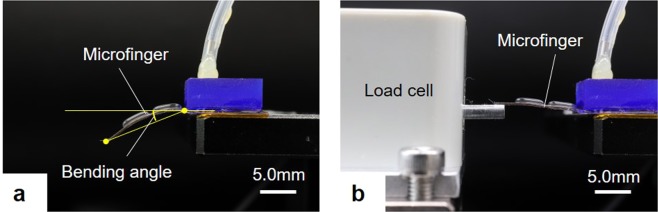
Experimental setup. The bending angle and generated force were evaluated in this study. (**a**) Experimental setup for bending angle measurement. The bending motion was observed from the side by a digital microscope (VHX-500F, KEYENCE Co.). The bending angle was defined by the line which links the tip and root of the microfinger as shown in (**a**). (**b**) Experimental setup for force measurement. The force was measured at the tip of microfinger by a load cell (LVS-5GA, Kyowa Electronic Instruments Co.) as shown in (**b**). The applied pressure for the evaluation of generated force was set at 70 kPa which drove and bent the PBA for the microfinger by 40°.

The bending motion of the microfinger was evaluated in terms of both the bending angle and the generated force. Figure 6a,b show the experimental setup for the measurement of bending angle and generated force. The bending motion was observed from the side by a digital microscope (VHX-500F, KEYENCE Co.). The magnification for the measurements in this experiment was 50. The bending angle was defined by the line that links the tip and root of the microfinger as shown in Fig. 6a. The force at the tip of the microfinger was measured with a load cell (LVS-5GA, Kyowa Electronic Instruments Co.), as shown in Fig. 6b. The applied pressure for a bending motion of 40° was estimated in advance and applied to the PBA in the experiments. Typically, the PBA for the microfinger in this study was bent by 40° at 70 kPa.

For the temperature sensor, the experimental setup for the measurement of the output voltage (Figs 2 and 3) with respect to the temperature involved a digital hot plate (DP-2S, AS ONE Co.) and a digital multimeter (34460 A, Keysight Tech. Inc.). The temperature was controlled by the digital hot plate and the output of the thermoelectromotive force was measured using a digital multimeter. The reference temperature was set to 20 °C in the experiments. In Fig. 3a, the temperature of the human fingertip was measured by developed thermocouple integrated into the microfinger. Digital thermometers (TX1003, Yokogawa Test & Measurement Co.) was used to confirm the temperature of the human fingertip at the same time. An evaluation setup for the characterization of the thermocouple depending on the bending motion consisted of a pneumatic regulator (ITV0050-3MS, SMC Co.) for pressure control and a digital multimeter (34460 A, Keysight Tech. Inc.). The microfinger was bent at the desired angle by the supplied pressure, which was controlled by the regulator. The microfinger was bent at a 40° angle by supplying approximately 70 kPa for characterization of the electrical resistance change (Figs 3 and 4). The electrical resistance of the thermocouple was measured using a digital multimeter (34460 A, Keysight Tech. Inc.).

## Data Availability

All data generated or analyzed during this study are included in this published article.
